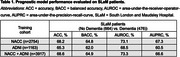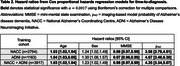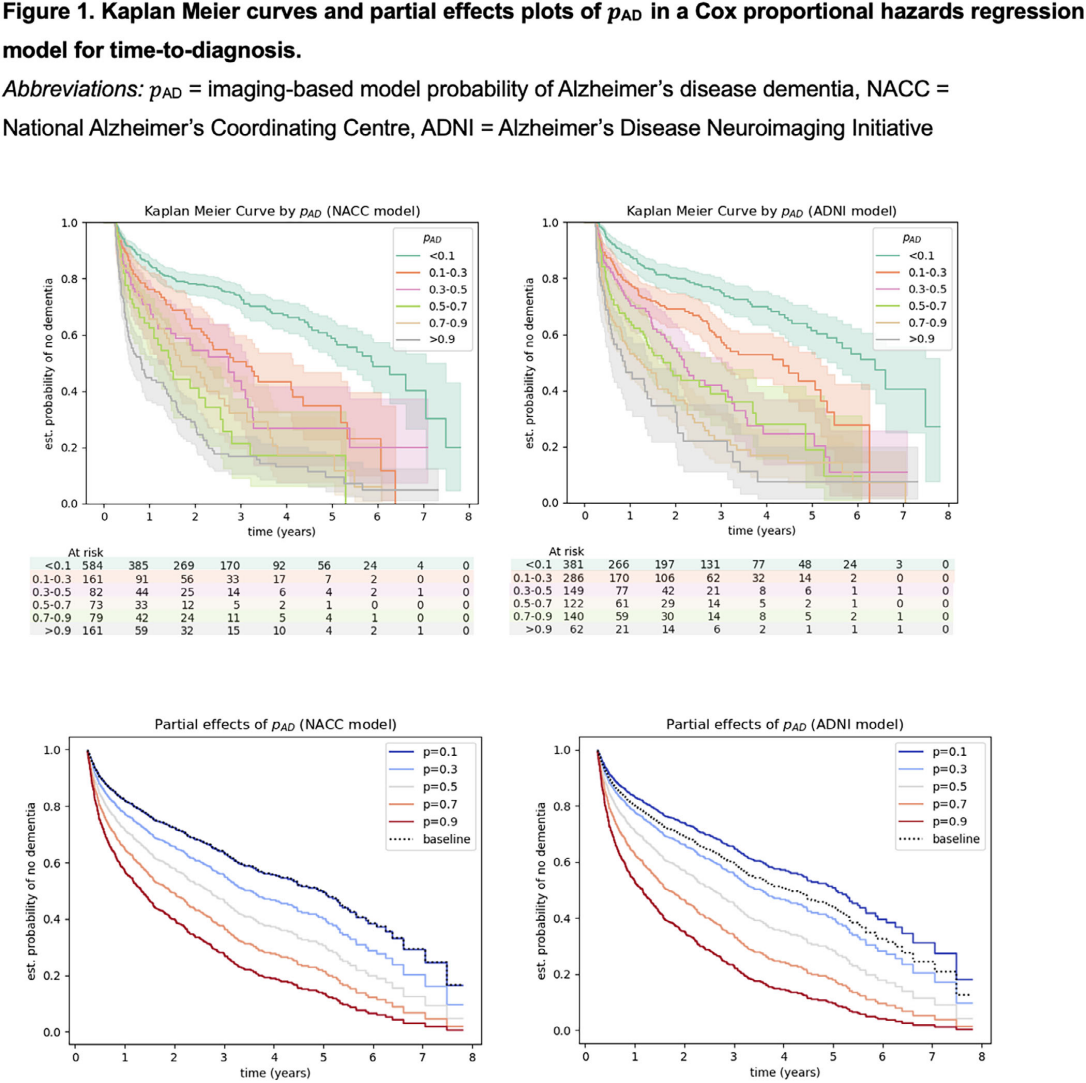# Neuroimaging‐based artificial intelligence for predicting dementia progression in a diverse real‐world memory clinic cohort

**DOI:** 10.1002/alz70856_102951

**Published:** 2025-12-26

**Authors:** Sophie A. Martin, Francesca Biondo, Amelia Jewell, Frederik Barkhof, James H. Cole

**Affiliations:** ^1^ UCL Hawkes Institute, University College London, London, United Kingdom; ^2^ Institute of Psychiatry, Psychology and Neuroscience, King's College London, London, United Kingdom; ^3^ South London and Maudsley NHS Foundation Trust, London, United Kingdom; ^4^ VU University Medical Centre, Amsterdam, Netherlands; ^5^ UCL Queen Square Institute of Neurology, University College London, London, United Kingdom

## Abstract

**Background:**

Existing applications of artificial intelligence for dementia prediction have largely relied on the use of large, curated research datasets for developing and evaluating model performance. Despite reported high accuracies, a significant gap remains in assessing their generalisability to real‐world data.

**Method:**

3D T1‐weighted magnetic resonance imaging scans were extracted from the South London and Maudsley Hospital (SLaM) NHS Trust and linked with de‐identified electronic health records via the Clinical Record Interactive Search database. This was used to identify 1140 individuals (self‐reported ethnicities: 19.7% Black/African/Caribbean, 11.0% Asian, 58.4% British/White, 10.4% Mixed/Other/Unknown) who received a scan at least 3 months prior to a dementia diagnosis or had a scan but no record of dementia (Biondo 2022, NeuroImage: Clinical). 3D ResNet models were trained using MRI from the National Alzheimer's Coordinating Center and the Alzheimer's Disease Neuroimaging Initiative, to classify individuals with Alzheimer's disease dementia from cognitively unimpaired individuals. First, the trained models were used to classify dementia patients from those with no dementia in the SLaM cohort using MRI only. Then, imaging‐based model probabilities of Alzheimer's disease dementia (p_AD_) were combined with covariates of age, sex and MMSE score in a Cox proportional hazards (Cox‐PH) regression model to estimate the time between the scan and recorded diagnosis. Data was right‐censored to account for individuals with no record of dementia.

**Result:**

Classification accuracies ranged from 65.3%‐68.6% as shown in Table 1. The hazard ratios for each covariate are summarised in Table 2. The p_AD_ hazard ratios [95% CI] were 3.58 [2.79,4.61], 4.67 [3.46,6.30] and 4.35 [3.34,5.67] for models trained on NACC, ADNI and both datasets respectively, and were all statistically significant (α=0.0017). Kaplan Meier curves and Cox‐PH partial effect plots for p_AD_ are shown in Figure 1.

**Conclusion:**

Models trained on large, research cohorts are effective at predicting progression to dementia for real‐world NHS patients, despite differences in the sample characteristics. p_AD_ was significant at estimating time‐to‐diagnosis, with a 0.1 increase in p_AD_ equating to a 13.6%‐16.7% increased risk of receiving a dementia diagnosis within 8 years. These findings demonstrate the clinical utility of generalisable artificial intelligence models in a real‐world context.